# Accuracy, Labor-Time and Patient-Reported Outcomes with Partially versus Fully Digital Workflow for Flapless Guided Dental Implants Insertion—A Randomized Clinical Trial with One-Year Follow-Up

**DOI:** 10.3390/jcm10051102

**Published:** 2021-03-06

**Authors:** Corina Marilena Cristache, Mihai Burlibasa, Ioana Tudor, Eugenia Eftimie Totu, Fabrizio Di Francesco, Liliana Moraru

**Affiliations:** 1 Department of Dental Techniques, Faculty of Midwifery and Medical Assisting (FMAM), “Carol Davila” University of Medicine and Pharmacy, 8 Eroilor Sanitari Blvd, 050474 Bucharest, Romania; mihai.burlibasa@umfcd.ro (M.B.); m_.tudor@stud.fim.upb.ro (I.T.); 2 Smart Biomaterials and Applications Master Program, Faculty of Medical Engineering, University Politehnica of Bucharest, 1-7 Gh. Polizu Street, 011061 Bucharest, Romania; 3 Department of Analytical Chemistry, Faculty of Applied Chemistry and Materials Science, University Politehnica of Bucharest, 1-7 Polizu St., Sector 1, 011061 Bucharest, Romania; 4 Multidisciplinary Department of Medical, Surgical and Oral Sciences, Campania University Luigi Vanvitelli (ex Second University of Naples), 6 Via Luigi De Crecchio, 80138 Napoli, Italy; fabrizio.difrancesco@unicampania.it; 5 Faculty of Dental Medicine, “Titu Maiorescu” University, 67A Gheorghe Petrascu Street, 040051 Bucharest, Romania; liliana.moraru@yahoo.com; 6 Oral and Maxillofacial Surgery Department, “Carol Davila” Central Military Emergency Hospital, 134 Plevnei Ave., 010825 Bucharest, Romania

**Keywords:** digital workflow, guided implant surgery, accuracy, flapless, digital impression

## Abstract

(1) Background: Prosthetically-driven implant positioning is a prerequisite for long-term successful treatment. Transferring the planned implant position information to the clinical setting could be done using either static or dynamic guided techniques. The 3D model of the bone and surrounding structures is obtained via cone beam computed tomography (CBCT) and the patient’s oral condition can be acquired conventionally and then digitalized using a desktop scanner, partially digital workflow (PDW) or digitally with the aid of an intraoral scanner (FDW). The aim of the present randomized clinical trial (RCT) was to compare the accuracy of flapless dental implants insertion in partially edentulous patients with a static surgical template obtained through PDW and FDW. Patient outcome and time spent from data collection to template manufacturing were also compared. (2) Methods: 66 partially edentulous sites (at 49 patients) were randomly assigned to a PDW or FDW for guided implant insertion. Planned and placed implants position were compared by assessing four deviation parameters: 3D error at the entry point, 3D error at the apex, angular deviation, and vertical deviation at entry point. (3) Results: A total of 111 implants were inserted. No implant loss during osseointegration or mechanical and technical complications occurred during the first-year post-implants loading. The mean error at the entry point was 0.44 mm (FDW) and 0.85 (PDW), *p* ≤ 0.00; at implant apex, 1.03 (FDW) and 1.48 (PDW), *p* ≤ 0.00; the mean angular deviation, 2.12° (FDW) and 2.48° (PDW), *p* = 0.03 and the mean depth deviation, 0.45 mm (FDW) and 0.68 mm (PDW), *p* ≤ 0.00; (4) Conclusions: Despite the statistically significant differences between the groups, and in the limits of the present study, full digital workflow as well as partially digital workflow are predictable methods for accurate prosthetically driven guided implants insertion.

## 1. Introduction

Prosthetically-driven implant positioning is a prerequisite for long-term successful treatment. The technological advancements in 3D medical imaging and the progresses registered by the use of Computer-Aided Design (CAD) and Computer-Aided Manufacturing (CAM) in dentistry make it possible to simulate ideal dental implants insertion, taking into consideration the available bone and neighboring tissues, without the need of opening a flap for direct anatomic structures visualization [1]. Transferring the planned implant position information to the clinical setting could be done using either static or dynamic guided techniques [2]. Dynamic guided surgery allows the operator to adjust in real time the implant positioning, based on previously acquired cone-beam computed tomography (CBCT) data, and perform implants insertion, at the planned position, with a navigation device equipped with an optical tracking system [3]. Static guided surgery requires the manufacturing of a custom-made template, reproducing the planned implants position [4]. Both techniques are clinically accepted, with good results. However dynamic guided surgery is less documented, and requires a significant initial investment and a certain learning curve [5,6]. Static guided surgery, on the other hand, is evidence-based and very popular due to the higher success rate and large accessibility, with a large amount of dedicated software available for accurate virtual implant planning, based on CBCT, scanned patient’s intraoral anatomy, and planned prosthetic restoration [1,7,8,9,10,11].

For static guided implant insertion, the surgical template, used for transferring the planed implant position to the surgical site, can be obtained using a partially or fully digital workflow. The partially digital workflow (PDW) includes one or more conventional laboratory steps during the implant planning phase [1], such as conventional impression of dental arches followed by digitalization of stone casts. With fully digital workflow (FDW), conventional impression is replaced with intraoral scanning of the anatomical structures. In both cases, DICOM (Digital Imaging and Communications in Medicine) files from CBCT and STL (standard tessellation language) files from the digital or digitalized impression are matched in the virtual planning software and the surgical template is designed based on planned final restoration.

In spite of the extended use of guided implant surgery, few randomized clinical trials (RCTs) evaluating its accuracy are available, and even fewer compared FDW to PDW [6,12].

Moreover, to our knowledge, no prior RCT compared FDW and PDW evaluating patient’s feedback and labor time from data collection to surgical template manufacturing.

The aim of the present randomized clinical trial (RCT) was to compare the accuracy of flapless dental implants insertion in partially edentulous patients with static surgical template obtained through PDW and FDW.

Patient outcome and time spent from data collection to template manufacturing were also compared.

The null hypothesis was that the accuracy of implant insertion using PDW does not statistically significantly differ from the use of FDW.

## 2. Materials and Methods

**Figure 1:**
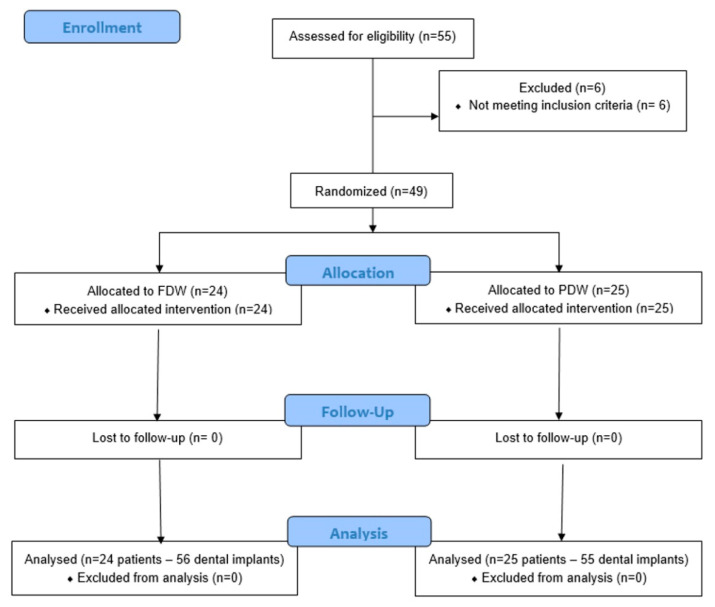
Consolidated Standards of Reporting Trials (CONSORT) flowchart [13]. From the 55 enrolled patients, 6 were excluded due to lack of meeting the inclusion criteria. A total of 49 patients with 66 edentulous sites were randomly assigned to one of the protocols, either fully digital workflow (FDW) or partially digital workflow (PDW) (33 sites for each protocol). A total of 56 implants were inserted in 24 patients with FDW and 55 implants were inserted in 25 patients with PDW. No loss to follow-up patients was registered at one-year evaluation period.

This study followed the Consolidated Standards of Reporting Trials (CONSORT) [13] criteria for improving the quality of reports of randomized trials, and was conducted between March 2019 and January 2021, in accordance with the ethical principles including the World Medical Association Declaration of Helsinki, the Belmont report, the Council for International Organizations of Medical Sciences (CIOMS) guidelines, and the International Conference on Harmonization in Good Clinical Practice (ICH-GCP). The study was approved by the Bioethical Committee of “Carol Davila” University of Medicine and Pharmacy (176/2018) and the protocol was registered with clinicaltrials.gov (accessed on 28 February 2021): ClinicalTrials.gov Identifier: NCT03814655. Written consent of each subject was also obtained. The CONSORT flow diagram is presented in Figure 1.

Inclusion criteria:

- Age over 18 years old, with good mental health and ability to fully understand and sign the consent form,
- Kennedy Class III partially edentulism with 4 or fewer missing teeth in a row,
- Good general health (healthy or with well-controlled chronical disease) with no contraindications for implant surgery,
- Acceptance of dental implant treatment and willing to comply with follow-up recalls,
- Acceptance of CBCT investigation.

Exclusion criteria:

- Parkinson’s disease (impossible to perform an accurate CBCT),
- Limited bone volume with stadial bone graft requirement,
- Limited mouth opening (impossible to use the surgical template),
- Untreated or uncontrolled periodontal disease,
- History of radiotherapy of the head and neck region,
- Poor oral hygiene and lack of compliance,
- Pregnancy or nursing.

### 2.1. Sample Size Calculation and Randomisation

Sample size calculation was conducted using mean (*SD*) of the angular deviation between planned and implant insertion, based on the results of Tallarico et al. [14] FDW, 1.43° (±1.98) and Varga et al. PDW [10], 3.04° (±1.51) in G*Power (Version 3.1.9.7 software, Heinrich-Heine University, Dusseldorf, Germany). The calculate effect size was 0.914 and the resulted sample size was 66 (33 implants sites for each protocol).

Each patient received panoramic radiography for initial screening and evaluation.

Randomization was performed using 66 opaque sealed envelopes with either FDW or PDW, 33 for each workflow, prepared and mixed by a member of the restorative team not involved in planning, surgery, or data collection. Patients were asked to pick an envelope after signing the consent form.

Each site could involve the insertion of 1 to 3 dental implants and each patient had no more than 4 implants sites randomly included in either FDW or PDW.

### 2.2. Data Collection

After initial examination the impression of the dental arch to be restored, the antagonists and centric occlusion are registered and the STL files are obtained:

- Digitally (FDW)—using Carestream 3600 (Carestream Dental LLC, Atlanta, GA, USA) intraoral surface scanner,
- Conventionally (PDW)—with condensation-cured polymethyl siloxane impression material (Speedex, Coltene, Switzerland), in two consistencies (two phase): putty and light body with Universal Activator and Coltene Adhesive in a stock tray. In the dental laboratory, a Type IV (Elite rock, Zhermack SpA, Polesine, Italy) dental stone was used for pouring all the models. To obtain the STL files, the models were digitalized with a D 700 3D scanner (3Shape, Copenhagen, Denmark).

For perfect 3D matching of the STL files of digital impression to the DICOM (Digital Imaging and Communications in Medicine) files of CBCT, a radiopaque tray (R2tray, MegaGen Implant, Daegu, Korea) was customized with polyether impression material (Impregum™ Penta™3M ESPE, St. Paul, MN, USA) on the dental arch selected for dental implants insertion.

A CBCT scan was performed for each patient with the customized R2tray, using ProMax 3D (Planmeca, Helsinki, Finland).

### 2.3. Design and Manufacturing of the Surgical Template

**Figure 2:**
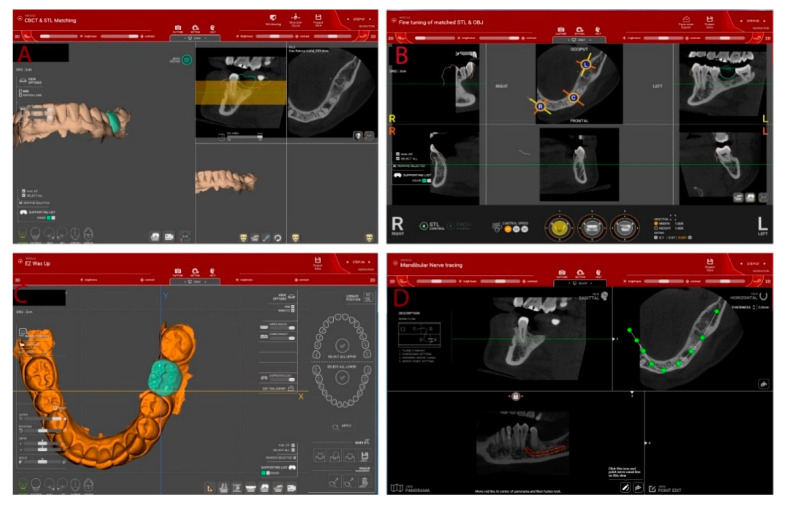
Dental implants insertion planning in R2GATE software. (**A**) Importing digital or digitalized impression (standard tessellation language (STL) files) and cone beam computed tomography (CBCT) Digital Imaging and Communications in Medicine (DICOM) files; (**B**) Superimposing files using the “best fit” algorithm; (**C**) Design of the prosthetic restoration (digital wax-up); (**D**) Marking the alveolar nerve trajectory.

All files (including scanned R2tray) in STL and DICOM format were imported in R2GATE version 2.0.0 (MegaGen, Daegu, Korea) and merged using the best-fitting repositioning tool. Manual fine adjustments were also performed (Figure 2). A digital wax-ul of the planned prosthetic restoration according to the functional and esthetic requirements was then designed in the same software.

**Figure 3:**
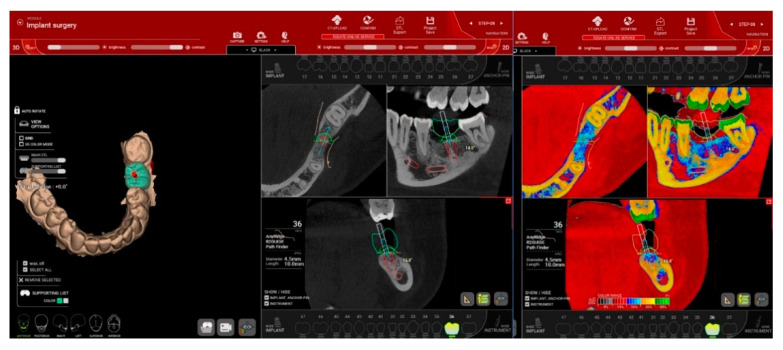
Planning implant directions, length and diameter in R2GATE software taking into consideration the final restoration and the available bone volume and quality. To facilitate bone quality assessment the “Digital Eye” option of the software (right) provides automatic conversion of CBCT gray scale in 5 basic colors, for bone density evaluation.

Implants’ positions as well as their length and diameter were then planned, taking into consideration the existing bone quantity and quality, the anatomical landmarks, and the final designed restoration (Figure 3). After performing the necessary changes, the treatment plan was approved, by the surgical team and the design of the surgical template with sleeve incorporated [4] was done in R2Ware version 1.10820 (MegaGen, Daegu, Korea). The surgical templates for all the implants sites were manufactured from e-shell^®^ 600 (Delta-Med GmbH, Freiburg, Germany), a light curing material to be used in a Digital Light Processing (DLP) additive manufacturing technology with EnvisonTEC Perfactory 3D printer (Gladbeck, Germany).

### 2.4. Dental Implants Surgery

The fit of the teeth supported surgical template was assessed intraorally prior to the surgery. All inserted implants were AnyRidge (MegaGen, Daegu, Korea). Surgical procedures were performed according to the manufacturer’s instructions, by one experienced surgeon (C.M.C.), under local anesthesia, using a flapless, minimally invasive approach. Before insertion, prophylactic antiseptic mouth rinse with 0.2% Chlorhexidine (Corsodyl, GlaxoSmithKline, Brentford, UK) was used for one minute to reduce bacterial contamination. The osteotomies sites were prepared according to the bone density, evaluated prior to the surgery on CBCT scans, using 3 parts (the stopper part, the guide part, and the drilling part) shank-modified drills [4,15]. All implants were inserted fully guided, using a hand ratchet up to the required landmark, for reproducing the planned insertion depth.

After implant insertion, digital impression was taken with the same intraoral scanner Carestream 3600 after screwing the corresponding 9 mm length scan abutments on the implants (AnyRidge, MegaGen, Daegu, Korea), for checking the position of the inserted fixtures. This registration of implant position was considered S1 for each inserted implant. All implants were covered with healing abutments and patients received written hygiene recommendations, including Chlorhexidine 0.2% mouth rinse and prophylactic antibiotic, 1 g of amoxicillin and clavulanate potassium, twice a day for the following 5 days. Non-steroidal anti-inflammatory (Ibuprofen 400 mg) was also prescribed for two days.

### 2.5. Prosthetic Procedures

Eight weeks postimplant insertion, new digital impression was taken using the same protocol and the implant position registration was considered S2 for each inserted implant. The final screw-retained zirconia crowns and bridges or customized zirconia abutments and cemented ceramic crowns for the esthetic zone, were delivered in 10 days to the patients and functionalized. Screw-retained crowns and bridges were screwed at 15 N/cm and abutments at 35 N/cm. One week post prosthesis insertion a panoramic radiograph was taken in all cases and patients were requested to evaluate through a Visual Analogue Scale (VAS) questionnaire their experience with the dental implant insertion and data collection. Follow-ups were scheduled at 6- and 12-months post prosthesis insertion. A panoramic radiograph at the 12-month follow-up was also performed.

**Figure 4:**
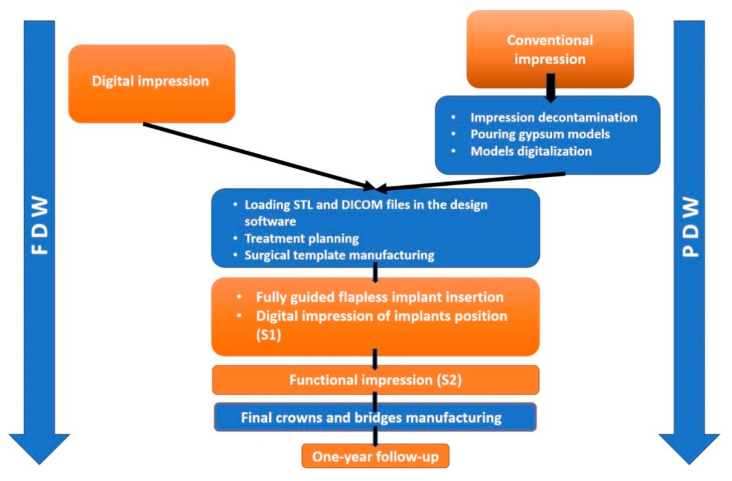
Study workflow: FDW—fully digital workflow, PDW—partially digital workflow including conventional impression, pouring models, models digitalization. Clinical steps are displayed in orange and laboratory steps are displayed in blue.

The fully digital and partially digital workflows are presented in Figure 4.

### 2.6. Outcome Measurements

All outcome measurements were performed by an independent *blind* researcher not knowing the protocol applied for the patients. The following parameters were evaluated:

#### 2.6.1. One-Year Implants Survival and Complications

Implants success was assessed according to Albrektsson et al. criteria [16] 1 to 4 (absence of mobility, radiographic absence of peri-implant radiolucency, peri-implant bone loss and absence of signs or symptoms as pain, infection, neuropathies, paresthesia). Implant mobility was evaluated with Osstell^®^ measurements (Osstell Mentor, Osstell, Goteborg, Sweden) at implant insertion and at 8 weeks, before initiating the prosthetic treatment. A percussion test was used for mobility evaluation at 6- and 12-months follow-up.

Periimplant marginal bone level was measured, at mesial and distal sites, on the panoramic radiographs post-implants insertion, at prosthetic insertion and at one-year post loading using Image J open software (imagej.nih.gov (accessed on 28 February 2021)), calibrated for each measurement based on the known implant length. For calibration with the known implant length, the reference points were implant apex and implant collar, and for mesial and distal bone level measurements the reference points were the correspondent horizontal point of the implant apex and the most coronal bone to implant contact. Bone level at implant insertion for each site was considered baseline. Bone loss at loading and one year follow-up was measure by subtracting the measured bone level from baseline for each site. The mean value for mesial and distal sites was recorded.

#### 2.6.2. Accuracy of Implants Insertion

Accuracy is defined in terms of precision and trueness (ISO 5725-2) [17,18]. Precision describes the degree of reproducibility between repeated measurements, and trueness describes the closeness of agreement to the object being measured [19,20]. 

Precision of measurements was evaluated by spatially comparing the post implant insertion intraoral scanning (S1) to the digital impression for the final prosthetic manufacturing (S2).

Assessment of trueness was performed by evaluating the deviation between planned (reference) and placed implants (S1), expressed by four deviation parameters.

STL files of the digital impression and treatment plan were imported in Exocad^®^ DentalCAD, version 2.3 Matera software (Exocad GmbH, Darmstadt, Germany) and the corresponding STL file of the inserted implant (length and diameter) was attached by matching the scan abutment using best fit algorithm.

**Figure 5:**
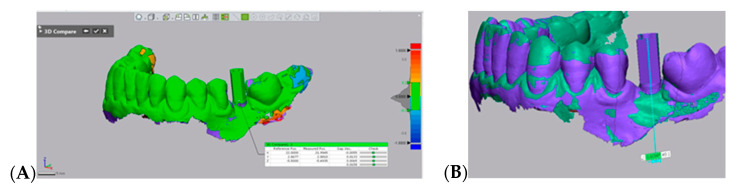
Precision assessment: S1 (digital scan at implant insertion) was compared with S2 (digital scan for final prosthetic manufacturing) in Geomagic Control X software. (**A**) 3D measurements at marginal border; (**B**) Angular deviation measurement.

Assessment of accuracy was done in Geomagic Control X software (3D Systems, Rock Hill, SC, USA) as follows:

- For precision evaluation S1 and S2 for each implant were compared at the level of the marginal border of the scan abutment (Figure 5A). S2 was set as reference and the 3D coordinate axes were defined (*x*: bucco-lingual, *y*: mesio-distal, and *z*: apico-coronally), and S1 was aligned to the reference based on best fit of the neighboring remaining teeth. Angular deviation was also evaluated (Figure 5B).
- For trueness evaluation S1 for each implant was 3D compared with the STL file of treatment plan. Treatment plan was set as reference and S1 was aligned based on the best fit of the neighboring teeth using the best fit algorithm of the software. To facilitate an accurate evaluation, irrelevant areas, beyond the field of interest, were removed [4].

**Figure 6:**
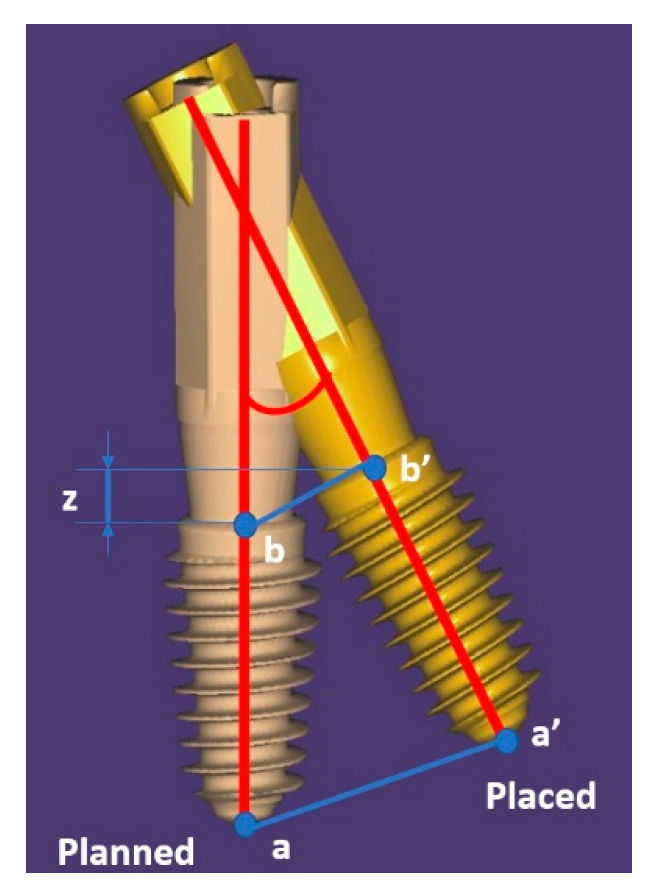
Trueness assessment was performed 3D at the implant entry point (b’ vs. b), at the implant apex (a’ vs. a), angular deviation between the axes of the planned and effectively placed implant, and vertical deviation on *z* axis.

For assessing trueness, the following parameters were recorded (Figure 6):

- 3D error at the entry point measured at the center of the implant (in mm),
- 3D error at the apex measured at the center of the implant apex (in mm),
- Angular deviation,
- Vertical deviation at entry point measured at the center of the implant (on *z*-axis).

**Figure 7:**
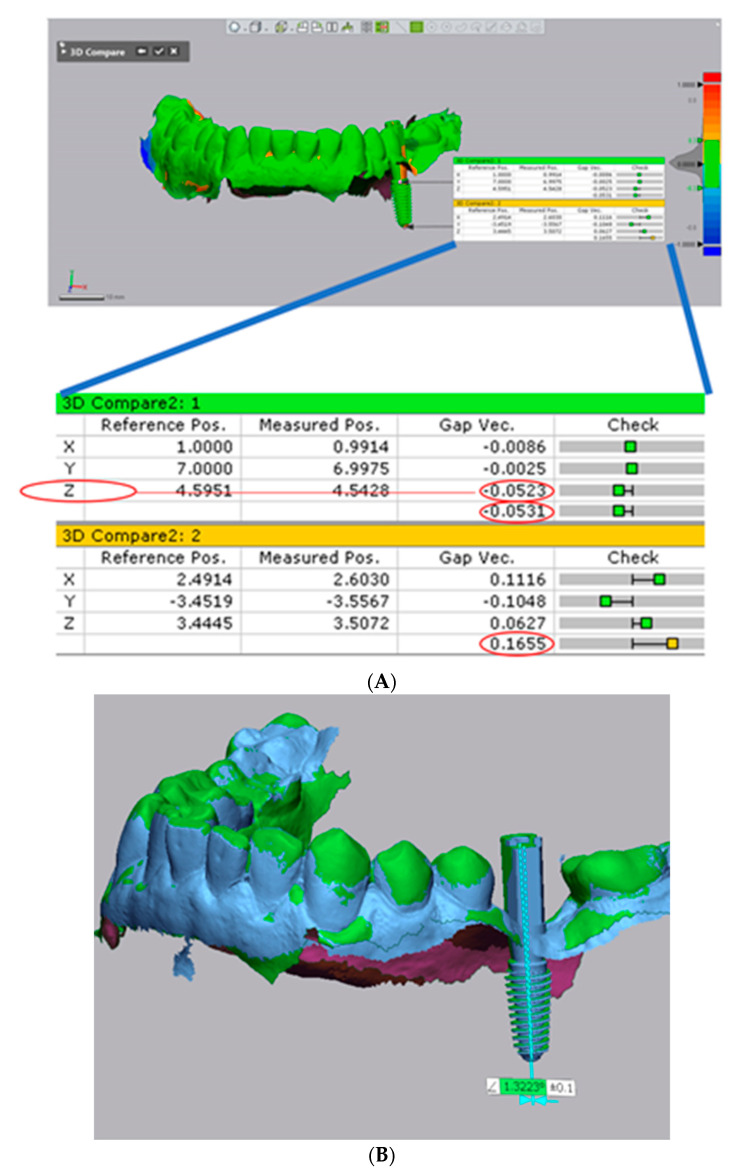
Trueness measurements in Geomagic Control X. (**A**) The displayed data were registered for assessment. (**B**) Measurement of angular deviation.

The 3D error was automatically calculated by the software taking into consideration the deviation on each direction set as follows: *x* = bucco-lingual error, *y* = mesio-distal error, and *z* = apico-coronally error, using Pythagorean theorem [21] and the absolute value was registered (Figure 7):

3D error = $\sqrt{x^{2}+y^{2}+z^{2}}$

#### 2.6.3. Patients’ Feedback Regarding Dental Implant Insertion

VAS, a psychometric response scale, can be utilized as a useful method to quantify, record, and evaluate qualitative outcomes that are difficult to measure by direct means [22]. The three VAS questions were stated as: “How was your experience with data collection (dental impression)? How was your experience with dental implants insertion? How do you describe your postoperative discomfort considering pain, swelling, bleeding?” [23]. Each question item was measured using a 10-point VAS, with 0 meaning not satisfied at all and 10 meaning completely satisfied.

#### 2.6.4. Labor Time from Data Collection to Dental Implants Surgery for FDW and PDW

For each workflow, the following data were recorded:

Clinical impression timing (conventional or digital), laboratory involved procedures from receiving clinical data to superimposing the files in design software.

### 2.7. Statistical Analysis

All data were synthetized in Excel tables, compared and analyzed. Origin Lab Pro 2019 (OriginLab Corporation, Northampton, MA, USA) was used for statistical analysis. Descriptive analysis was performed for numerical values and mean, SD with 95% confidence interval (95% CI) were calculated. Mann–Whitney *U* nonparametric test was used to assess statistically significant differences. Significance was set at a *p*-value < 0.05.

## 3. Results

Fifty-five patients were assessed for eligibility, 6 patients did not comply with the inclusion criteria, and 49 patients were enrolled in the present study, with 66 edentulous sites, and a total number of 111 implants were inserted. The characteristics of the implants and patients are presented in Table 1. All surgery were fully guided (the guide provided physical guidance up to the insertion of the implant) and flapless (no open flap or sutures were required).

### 3.1. One Year Implant Survival and Complications

No implant loss during osseointegration or first year of function was recorded. ISQ (implant stability quotient) values measured with Osstell Mentor^®^ at prosthetic procedures initiation did not decreased as compared to implant insertion. No dropouts were registered at one-year follow-up period. No mechanical and technical complications occurred during the first-year post-implants loading.

The mean bone loss at prosthetic insertion and one-year follow-up is presented in Table 2.

### 3.2. Accuracy of Implant Insertion

#### 3.2.1. Assessment of Precision

Reproducibility assessment of postoperative intraoral scanning (S1) and digital impression for prosthetic manufacturing (S2) did not register statistically significant differences for FDW and PDW. The mean values and SD at the measured at the vestibular border of the scan abutment and angular deviation are presented in Table 3.

No statistical significant difference was registered between the measurements in FDW and PDW group.

#### 3.2.2. Assessment of Trueness

A mean of 0.44 mm for FDW and 0.85 mm for PDW was registered at the implant’s entry point with a maximum of 0.98 mm for FDW and 2.30 at PDW.

The mean error at the implant apex was 1.03 mm for FDW and 1.48 mm for PDW (Table 4). A mean angular deviation of 2.12° and 2.48° was registered for FDW and PDW, respectively. The mean vertical deviation (on z axis) at the entry point was also greater at PDW comparing to FDW, 0.68 mm, respectively 0.45 mm. All the P values showed statistically significant differences between FDW and PDW (Table 4).

A statistically significant difference for all assessed parameters is observed also when maxilla and mandible are analyzed separately.

**Figure 8:**
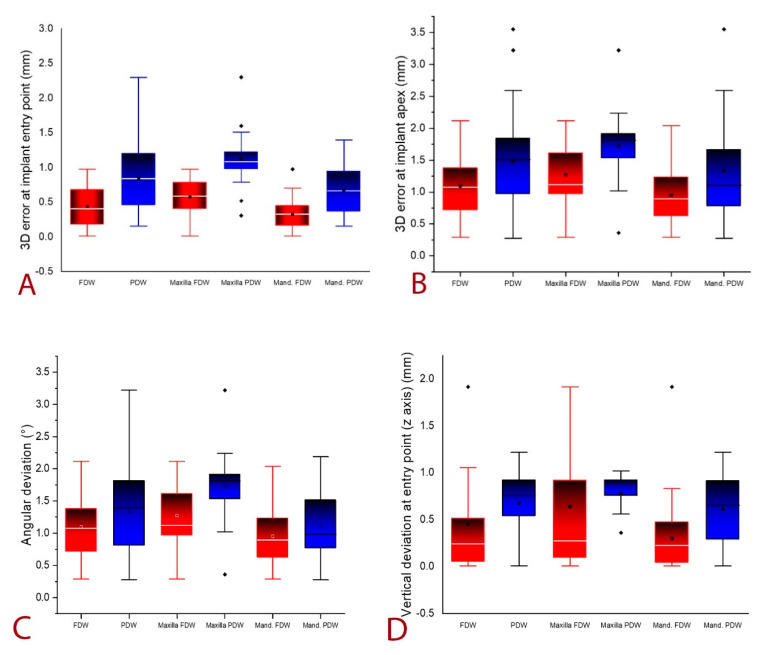
(**A**) 3D error at implant entry point overall and separately evaluated for maxilla and mandible; (**B**) 3D error at implant apex overall and separately evaluated for maxilla and mandible. (**C**) Angular deviation; (**D**) Vertical deviation at entry point. The boxplots symbols significance: box—interquartile range (IQR), ᵻ range within IQR, — median line, □ mean, ◊ outliners. Red plots are for FDW group and blue plots are for PDW group.

The overall errors registered at the assessed landmarks as well as for the angular deviation between planned and placed implants for FDW (red plots) and PDW (blue plots) and the separate evaluation for maxillary and mandibular implants were presented in Figure 8A–D.

As it can be observed, mandibular inserted implants with FDW registered the highest insertion trueness.

### 3.3. Patients’ Feedback Regarding Dental Implant Insertion

**Figure 9:**
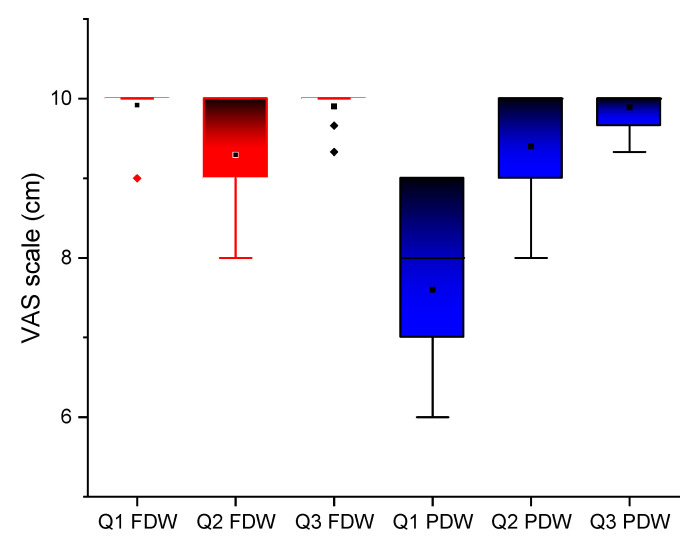
Assessment of response to the VAS questionnaire (0 meaning not satisfied at all and 10 meaning completely satisfied): Q1: How was your experience with data collection (dental impression)?; Q2: How was your experience with dental implants insertion?; Q3: How do you describe your postoperative discomfort considering pain, swelling, bleeding? The boxplots symbols significance: box—interquartile range (IQR), ᵻ range within IQR, — median line, □ mean, ◊ outliners. Red plots are for FDW group and blue plots are for PDW group. Most of the patients in FDW grouped scored 10 at Q1 (minimum 9) comparing to PDW (scores between 6 and 9). Dental implants insertion and postoperative patient’s feedback was favorable, for both groups (VAS score between 8 and 10).

The results of VAS questionnaires are presented in Figure 9.

A statistically significant difference *p* ≤ 0.00 was registered for the first question: “How was your experience with dental implants insertion?” A minimum value of 6 was noticed for PDW and 9 for FDW. However, a better general score, meaning better patient’s feedback, was noticed for FDW. For the following two questions, “How was your experience with dental implants insertion?” and “How do you describe your postoperative discomfort considering pain, swelling, bleeding?’’ registered high scores (between 8 and 10) with no statistical significance between the two protocols, *p* = 0.42 and 0.54, respectively. For implants insertion and postoperative wound healing, patients had positive experiences with both protocols.

### 3.4. Labor Time from Data Collection to Dental Implants Surgery for FDW and PDW

The time spent for each clinical and laboratory step was rounded to the next minute and displayed in Table 5.

A considerably longer time is required in PDW due to the necessity of clearing, decontamination of the impressions, pouring the models, and scanning with the laboratory scanner for obtaining the digital files. Also 60 min were registered as mean timing for sending the package. However, depending on day or traffic, this could take longer.

## 4. Discussion

The present RCT was conducted to evaluate the fully digital and the partially digital workflow, the latter including at least one or more conventional laboratory steps during the implant planning phase. Our aim was to assess the accuracy of guided implant placement as well as patients’ feedback regarding data collection, surgery, and postoperative wound healing. The time spent from the impression taking, either digital or conventional, to the treatment plan was also evaluated.

To avoid bias, all implant surgeries were performed by a single operator, not knowing about the workflow protocol. Also, the same operator performed postoperative intraoral scanning for all patients.

The null hypothesis was partially rejected. For assessing accuracy of implants insertion, precision and trueness were separately evaluated. Precision, determined by comparing postoperative and functional digital impression, did not statistically significant differ in PDW from FDW (Table 3). On the other hand, trueness, comparing the planned with the effectively inserted implant was statistically significantly different for the two protocols when it was globally analyzed and separately for maxilla and mandible. FDW lead to implants placed closer to the planned position, less 3D error at entry point, apex, less vertical deviation as well as less angular deviation compared to PDW (Table 4, Figure 8A–D).

To the authors’ knowledge, there are, to date, two published RCT assessing full and partially workflow for dental implants insertion but none evaluated patient-reported outcomes or time spent for data collection to design the prosthetically driven surgical template. Moreover, the first RCT published in 2019 by Tallarico et al. included 20 patients (10 in each group) and 57 implants inserted with both tooth-supported and tooth-mucosal-supported surgical templates, anchored with pre-planned pins, and the implants insertion was flapless or flap-open [8]. The implants insertion accuracy was evaluated in Dental SCAN v6 software by comparing postoperative STL with planned treatment and the angular deviation was calculated taking into consideration implants length and diameter. The authors found no statistically significant differences between full and partially digital workflow with a higher discrepancy in vertical plan, probably, according to the authors, due to the distortion of digital impression in cases of large edentulous areas [8]. Our study was limited to Kennedy Class III partially edentulism with four or fewer missing teeth in a row, exclusively with a tooth-supported surgical template, all surgery being performed flapless. These limits set as inclusion criteria could explain the statistically significantly better accuracy of the full digital workflow in the present study.

On the other hand, in the second RCT on fully digital versus partial digital workflow, Kiatkroekkrai et al. performed static guided surgery on 47 patients, receiving 60 single implants. The authors evaluated accuracy on postoperative CBCT, by comparing planned and placed implants in coDiagnostiX software [9] and found a lack of statistical significance but with tendency towards a smaller deviation when the intraoral scanner was used. The difference could be due to the larger sample size of our study, 110 implants inserted in 66 edentulous sites, 33 for each protocol.

In the present study both implant insertion protocols achieved successful results at one-year follow-up, with no complications and reduced periimplant bone loss, with not statistically significant difference between FDW and PDW for implants inserted in maxilla as well as in mandible (Table 2). Moreover, bone gain was noticed at one-year follow-up for 15 implants (six in the FDW group and nine in the PDW group). This could be explained by the type of implant used, with conical Morse taper connection [24,25], with platform switching connection implant-abutment [26,27] and the flapless surgery with preserving the periosteal blood support [28].

The accuracy of implants insertion was assessed according ISO 5725-2 [18] in terms of precision and trueness. Precision, or reproducibility, was evaluated by comparing the two intraoral scanning, at implants insertion (S1) and functional digital impression (S2). No significant difference was noticed between the two groups and the obtained values were in accordance with other studies comparing the accuracy of different intraoral scanners [29].

For trueness evaluation, planned and placed implants were 3D compared. Two different methods, radiographic and non-radiographic, can be used to compare placed with planned implant position [11]. Tang et al. [30], comparing both methods, found no significant difference between the digital registration method and the radiographic method in evaluating the clinical accuracy of the implant. Moreover, the radiographic method requires a postoperative CBCT with further radiation exposure [4]. In our study, the digital registration method was used with the metrology software program Geomagic Control X were the models with planned and effectively placed implants were superimposed using the “best fit” scenario, the planned file being set as reference. In this reverse engineering program, the function “best fit” can align two similar 3D models by the size of the point cloud (sample size). Following the alignment, the software automatically calculates the 3D error on the requested landmark (Figure 7A). Angular deviation was determined between the central axes of the two compared files (Figure 7B). Four recommended parameters were assessed in the present study [31]: 3D deviation at the entry point, 3D deviation at the implant apex, deviation of the long axis (angular deviation), and deviation in height/depth (on z axis). In spite of the statistically significant differences between the two protocols (FDW and PDW), the placement errors measured are in accordance with the existing literature [1,32,33]. A systematic review with meta-analysis of 13 clinical studies by Siqueira et al. [1] on 669 implants inserted in 325 patients revealed a global coronal deviation of 1.03 mm (95% CI: 0.88–1.18 mm), mean global apical deviation of 1.33 mm (95% CI: 1.17–1.50 mm), mean angular deviation of 2.68° (95% CI: 2.32°–3.03°), and mean depth deviation of 0.59 mm (95% CI: 0.46–0.70 mm). Another systematic review of 14 clinical studies by Zhou et al. [33] reported a mean global deviation of 1.25 mm at the implant platform, 1.57 mm at the implant apex, and a mean angular deviation of 4.1°. A systematic review of 24 clinical and preclinical studies by Tahmaseb et al. [21] revealed mean total errors of 1.12 mm at the implant platform and 1.39 mm at the implant apex, and a mean angular deviation of 3.89°. The mean error in our study was 0.44 mm at the entry point (implant platform) for FDW and 0.85 for PDW, similar to the results obtained by Lin et al. [34] (0.78 mm), Cristache and Gurbanescu 0.79 mm [4] and Kiatkroekkrai et al. 0.87(full digital group) and 1.01 (partially digital group) [9].

At the implant apex, the mean errors measured in our study were 1.03 for FDW and 1.48 for PDW, comparable to other studies: Lin et al. [34], 1.28 mm, Cristache and Gurbanescu 1.17 mm [4], Kiatkroekkrai et al., 1.10 for full digital group and 1.38 for partially digital group [9].

The mean angular deviation was 2.12° (FDW) and 2.48° (PDW), similar to Kiatkroekkrai et al. [9]; 2.42°, Tallarico et al., 2.20° [8]; Cristache and Gurbanescu, 2.34°; but lower than the mean value obtained by Lin et al. [34], 4.30°.

The mean depth deviation (on z axis) was 0.45 mm for the FDW group and 0.68 mm for PDW, different to the results obtained by Tallarico et al. 0.58 (±0.44) for the full digital group and 0.46 (±0.34) for the partially digital group [8]. The higher vertical deviation for the full digital group was probably due, according to the authors, to the tilting of the surgical template during the drilling for long span edentulous area.

However, the general obtained results, were in the safety limits for both protocols (FDW and PDW), and the better accuracy of this study is probably due also to the use of a surgical template with sleeve incorporated in the design and shank-modified drills for minimizing mechanical tolerance of the instruments, with no need for additional metal sleeves [4,35,36].

The present study VAS 10 cm scale was used for patient-reported outcome measures regarding the treatment. A statistically significant positive outcome was registered by the FDW group at the first question on data collection for treatment planning (digital versus conventional impression). This result was probably due to a better tolerance of intraoral scanning procedure comparing to the conventional impression technique causing gag reflex, discomfort and even pain to some patients [37]. The lower score registered on conventional impression was also due to the comparison made by the patients between the two data registration methods. The two other questions regarding the surgery and postoperative outcomes did not registered differences between the groups.

Another aspect assessed was the time spent to collect the information required for the surgical template manufacturing. 17 to 21 min are necessary if FDW is adopted, comparing to 148–151 min for PDW. Moreover, with FDW cross-infection contamination is limited due to direct transfer of virtual information.

The obtained result need to be interpreted with caution. All surgeries were performed by a single experienced surgeon, well knowing the dental implant system who adjusted the drilling protocol to the bone density. It will be usefully to assess accuracy of guided dental implants insertion with less experienced practitioners.

Also, the results could be different with the use of a tooth-mucosal or just a mucosal-supported surgical template.

## 5. Conclusions

The aim of the present RCT was to compare the accuracy of flapless dental implants insertion in partially edentulous patients, with static surgical template obtained through PDW and FDW, and to assess implants survival and complications at one-year follow-up. Patient outcome and time spent from data collection to template manufacturing were also compared. The stated null hypothesis was that the accuracy of implant insertion using PDW will not statistically significantly differ from the use of FDW.

Accuracy was judged, according to ISO 5725-2, for precision, by comparing postoperative and functional digital impression, and trueness, by non-radiographic evaluation of planned and placed implants.

Patient outcomes were evaluated using VAS questionnaire.

The null hypothesis was partially rejected. A statistically significant better trueness was registered at the entry point, at the apex, in angular deviation, and on the z axis (vertical deviation) at the entry point for the FDW group compared to the PDW group, but within the safety limits for all cases. Also, patients scored statistically significantly better the impression method for FDW, and the time spent for data collection and processing to surgical template design was shorter for the FDW group comparing to the PDW group.

To our knowledge, this was the first RCT comparing patients’ feedback and time spent for data collection to design the surgical template with FDW and PDW, in partially edentulous patients, with both mesial and distal tooth support. It will be interesting to investigate in future studies if, for tooth-mucosal or just mucosal supported surgical template, the FDW will be as accurate.

Within the limitation of the present RCT, full digital workflow as well as partially digital workflow are predictable methods for accurate prosthetically driven guided implants insertion.

## Figures and Tables

**Table 1:** Characteristics of evaluated groups.

Characteristics	FDW	PDW
Number of patients	24	25
Mean age at implant insertion (SD)	54.45 (±11.11)	54.58 (±9.41)
Gender (F/M)	14/10	18/7
Number of restored sites	33	33
Number of implants 111 (maxilla/mandible)	56 (25/31)	55 (21/34)
Single/multiple gap	12/21	14/19
Implants length (mean, SD) min/max (mm)	10.05 (±1.40) 7/13	9.80 (±1.29) 8.5/13
Implants diameter (mean, SD) min/max (mm)	3.91 (±0.43) 3.5/4.5	4.06 (±0.39) 3.5/4.5

FDW—fully digital workflow, PDW—partially digital workflow, SD—standard deviation.

**Table 2:** Mean bone loss at prosthetic insertion and one-year follow-up.

Bone Loss (mm) Mean [SD ± (95% CI)]	Loading		*p* Value	1 Year Follow-Up		*p* Value
	FDW	PDW		FDW	PDW	
Maxilla	0.04 [±0.15 (−0.02–0.10)]	0.10 [±0.13 (−0.05–0.07)]	0.13	0.05 [±0.12 (−0.00–0.10)]	0.11 [±0.12 (0.05–0.17)]	0.08
Mandible	0.07 [±0.11 (0.03–0.11)]	0.04 [±0.12 (0.00–0.08)]	0.22	0.03 [±0.13 (−0.01–0.08)]	0.01 [±0.14 (−0.04–0.06)]	0.59
Overall	0.06 [±0.13 (0.03–0.09)]	0.07 [±0.13 (0.04–0.10)]	0.93	0.04 [±0.12 (0.01–0.07)]	0.05 [±0.14 (0.01–0.09)]	0.55

The negative values meaning bone gain. FDW—fully digital workflow, PDW—partially digital workflow, SD—standard deviation, CI—confidence interval.

**Table 3:** Assessment of precision.

S1 vs. S2	FDW Mean (SD)	PDW Mean (SD)	*p* Value
3D errors at vestibular border (abutment) (mm)	0.042 (±0.013)	0.044 (±0.016)	0.23
Angular error (°)	0.264 (±0.085)	0.270 (±0.100)	0.95

FDW—fully digital workflow, PDW—partially digital workflow, SD—standard deviation.

**Table 4:** Assessment of trueness for FDW and PDW.

	FDW (mm) Mean [SD± (95% CI)] min/max	PDW (mm) Mean [SD± (95% CI)] min/max	*p* Value
3D error entry point (mm)—overall	0.44 [±0.28 (0.36–0.51)] 0.01/0.98	0.85 [±0.43 (0.73–0.96)] 0.16/2.30	*p* ≤ 0.00
3D error entry point (mm)—maxilla	0.58 [±0.30 (0.46–0.70)] 0.01/0.98	1.12 [±0.40 (0.94–1.30)] 0.31/2.30	*p* ≤ 0.00
3D error entry point (mm)—mandible	0.33 [±0.22 (0.25–0.41)] 0.01/0.98	0.68 [±0.35 (0.55–0.80) 0.16/1.40	*p* ≤ 0.00
3D error apex (mm)—overall	1.03 [±0.48 (0.97–1.22)] 0.29/2.12	1.48 [±0.72 (1.29–1.68)] 0.28/3.55	*p* ≤ 0.00
3D error apex (mm)—maxilla	1.27 [±0.47 (1.08–1.47)] 0.29/2.12	1.72 [±0.53 (1.48–1.97)] 0.36/3.22	*p* ≤ 0.00
3D error apex (mm)—mandible	0.95 [±0.43 (0.79–1.10)] 0.29–2.04	1.33 [±0.78 (1.06–1.60)] 0.28/3.55	0.04 *
Angular deviation (°)—overall	2.12 [±0.85 (1.90–2.35)] 0.39/3.86	2.48 [±0.75 (2.27–2.68)] 1.15/3.95	0.03 *
Angular deviation (°)—maxilla	2.5 [±0.73 (2.20–2.80)] 0.39/3.50	2.92 [±0.57 (2.66–3.18)] 1.28/3.95	0.04 *
Angular deviation (°)—mandible	1.82 [±0.83 (1.52–2.12)] 0.71/3.86	2.20 [±0.72 (1.95–2.45)] 1.15/3.91	*p* ≤ 0.00
Vertical deviation at entry point (z axis) (mm)—overall	0.45 [±0.57 (0.30–0.60)] 0.01/1.92	0.68 [±0.28 (0.60–0.75)] 0.01/1.22	*p* ≤ 0.00
Vertical deviation at entry point (z axis) (mm)—maxilla	0.64 [±0.70 (0.36–0.93)] 0.01/1.92	0.78 [±0.17 (0.80–0.86)] 0.36/1.02	0.01 *
Vertical deviation at entry point (z axis) (mm)—mandible	0.30 [±0.38 (0.16–0.44)] 0.01/1.92	0.61 [±0.32 (0.50–0.72) 0.01/1.22	*p* ≤ 0.00

* Statistically significant *p* < 0.05, FDW—fully digital workflow, PDW—partially digital workflow, SD—standard deviation, CI—confidence interval.

**Table 5:** Labor time for data collection and processing.

	FDW (in min.)	PDW (in min.)
Impression two arches and occlusion registration (clinic)	8–12 5 (R2tray digitalization)	9–12
Data sent to the lab	2 (data transfer)	5 (cleaning & decontamination) 2 (packing) 60 (sending package)
Laboratory impression decontamination and check	-	5
Pouring gypsum models	-	45
Casts digitalization	-	15 (arch model scanning) 5 (R2tray scanning)
Loading STL files in the design software	2	2
TOTAL	17–21	148–151

FDW—fully digital workflow, PDW—partially digital workflow.
